# Neutralizing Antibody Responses in Macaques Induced by Human Immunodeficiency Virus Type 1 Monovalent or Trivalent Envelope Glycoproteins

**DOI:** 10.1371/journal.pone.0059803

**Published:** 2013-03-22

**Authors:** Gerald V. Quinnan, Pengfei Zhang, Ming Dong, Hong Chen, Yan-Ru Feng, Mark Lewis, Christopher C. Broder

**Affiliations:** 1 Uniformed Services University of the Health Sciences, Bethesda, Maryland, United States of America; 2 Bioqual, Inc., Rockville, Maryland, United States of America; Shanghai Medical College, Fudan University, China

## Abstract

A major goal of efforts to develop a vaccine to prevent HIV-1 infection is induction of broadly cross-reactive neutralizing antibodies (bcnAb). In previous studies we have demonstrated induction of neutralizing antibodies that did cross-react among multiple primary and laboratory strains of HIV-1, but neutralized with limited potency. In the present study we tested the hypothesis that immunization with multiple HIV-1 envelope glycoproteins (Envs) would result in a more potent and cross-reactive neutralizing response. One Env, CM243(N610Q), was selected on the basis of studies of the effects of single and multiple mutations of the four gp41 glycosylation sites. The other two Envs included R2 (subtype B) and 14/00/4 (subtype F), both of which were obtained from donors with bcnAb. Rhesus monkeys were immunized using a prime boost regimen as in previous studies. Individual groups of monkeys were immunized with either one of the three Envs or all three. The single N610Q and N615Q mutations of CM243 Env did not disrupt protein secretion, processing into, or reactivity with mAbs, unlike other single or multiple deglycosylation mutations. In rabbit studies the N610Q mutation alone or in combination was associated with an enhanced neutralizing response against homologous and heterologous subtype E viruses. In the subsequent monkey study the response induced by the R2 Env regimen was equivalent to the trivalent regimen and superior to the other monovalent regimens against the virus panel used for testing. The 14/00/4 Env induced responses superior to CM243(N610Q). The results indicate that elimination of the glycosylation site near the gp41 loop results in enhanced immunogenicity, but that immunization of monkeys with these three distinct Envs was not more immunogenic than with one.

## Introduction

Induction of highly potent, bcnAb is a major goal of current efforts to develop a vaccine for prevention of Human Immunodeficiency Virus Type 1 (HIV-1) infections. This goal is made difficult by the remarkable neutralization resistant nature of the virus strains commonly found in infected people. Nevertheless, there is clear evidence that humans can develop antibody responses that are highly effective in neutralizing HIV-1. This evidence includes extensively cross-reactive neutralizing antibody responses that develop in some cases of HIV-1 infection [1]–[8], and the cross-reactive neutralizing activity of certain human monoclonal antibodies (mAbs) obtained from infected people [9]–[33].

Neutralization resistance of HIV-1 has been attributed to a variety of factors, including masking of neutralization epitopes by glycans and surface loop structures, and a global neutralization resistance phenotype [13], [34]–[37]. Moreover, certain epitopes may be expressed only transiently during the fusion process providing little opportunity for antibody binding. This phenomenon of transient epitope expression has been termed “conional masking” by Kwong [38]. Such conformational masking may distinguish Tier 1 (neutralization sensitive) and Tier 2 (neutralization resistant) viruses.

Combinations of antibodies with multiple specificities may overcome the neutralization resistance of HIV-1 primary isolates [5], [8]. Pooled human IgG from HIV-1 infected donors commonly neutralizes many strains of HIV-1, and can protect monkeys immunized passively against experimental challenge with Simian-Human Immunodeficiency Virus (SHIV) [39]. The potential value of antibodies against multiple specificities in protection against infection has been further demonstrated in passive immunization studies using human mAbs. Administration of a combination of the cross-reactive mAbs 2F5, IgG1b12, and 2G12 to monkeys is protective against SHIV challenge [40]–[43].

A variety of innovative approaches have been tried for induction of primary virus neutralizing antibodies using HIV-1 Env-based immunogens, but none has resulted in induction of neutralizing antibodies that are both highly potent and highly cross-reactive against Tier 2 viruses. Our laboratory has studied a variety of approaches to Env-based immunization which have generated variably positive neutralizing responses. One approach that we have used has involved administration of alphavirus replicon particles expressing HIV-1 Env in a primary immunization series, followed by administration of recombinant soluble gp140 Env in adjuvant as a booster immunization. These immunogens have been tested in mice, rabbits, and monkeys in efforts to optimize neutralizing responses. Work reported to date has involved use of an Env designated strain R2, from an individual, referred to elsewhere as FDA2, with broadly cross-reactive neutralizing antibodies (bcnAb) [44]–[47]. Immunization of monkeys with the R2 Env using the alphavirus prime - gp140 in RiBi adjuvant booster immunization regimen resulted in neutralizing responses that cross-reacted with 13/17 HIV-1 strains tested and protection of some animals from infection by heterologous SHIV challenge [45]. Nevertheless, protection against infection was limited to monkeys with the highest neutralization titers and cross-reactivity against other SHIV strains was very limited. More recently, we have reported the induction in rabbits of extensively cross-reactive neutralization of well-established Tier 2 strains of varied subtypes, using R2 gp140 in an adjuvant produced by GlaxoSmithKline Biologicals for immunization [48]. The effect of this regimen in nonhuman primates is under evaluation. Despite the extensive nature of the cross-reactivity of the rabbit responses, the magnitude of the responses was not of the level that would be anticipated to result in protection of monkeys against SHIV challenge based on our earlier findings. Immunization regimens that result in either more cross-reactive or more potent responses than we have achieved to date are needed.

To date, the precise Env characteristics that are important for use as successful immunogens remain ill defined. The R2 Env is from a donor with bcnAb and is highly unusual with respect to its capacity to infect cells in the absence of CD4 [47]. Its immunogenicity is also unusual in that other Env immunization regimens have not induced immunity against heterologous SHIV challenge, and administration of the YU2 strain Env in the same GSK adjuvant did not result in the extensive cross-reactivity observed in our study [49]. Thus, it appears that some Envs may be more immunogenic than others, and it is possible that the presence of a bcnAb response in an individual may reflect certain characteristics of the virus strain with which they are infected. In addition, we have studied the characteristics of Envs from a number of individuals followed in a cohort study at the Institute for Tropical Medicine in Antwerp, who were observed to have bcnAb [50]. Common characteristics among these Envs were high sensitivity to neutralization by mAbs directed against the CD4 binding site and membrane proximal epitope region (MPER) in gp41. Interestingly, Env 14/00/4, from one of these patients, displayed high sensitivity to neutralization by the 2F5 mAb that was dependent upon an extremely rare 2F5 epitope sequence, and that also conferred sensitivity to neutralization by sCD4, an anti-CD4bs mAb, and sera from HIV-1 infected individuals ([50]and unpublished). We hypothesize that this rare mutation in 14/00/4 and the unique V3 sequence of R2 that causes CD4-independence, both cause critical cross-reactive neutralization epitopes to be conformationally unmasked on the proteins, such that they are capable of inducing exceptional neutralizing antibody responses. Thus, an examination of the comparative neutralizing responses to R2 and 14/00/4 gp140 s could help define the nature of these two individual cases of bcnAb responses, and this was a goal of the present study.

A second theme presented in this study is characterization of the immunogenicity of gp140 s derived from varied HIV-1 subtypes. The induction of antibodies that neutralize CRF1 (subtype A/E) strains has also been an elusive goal. Indeed, even a wild-type CRF1 gp140 tested in the present study did not induce homologous neutralizing antibodies, whereas, the same Env with one particular gp41 deglycosylation mutation was found to induce an enhanced neutralizing antibody response. The actual target epitopes exposed as a result of deglycosylation are unknown, but it is likely that the effect of deglycosylation on immunogenicity of the CRF01_A/E strain occurs by a different mechanism than any effect of V3 or MPER mutations might have on immunogenicity of the R2 or 14/00/4 strains. Given that R2 is subtype B, 14/00/4 is subtype F, and CM243 is subtype CFR01_A/E, and that the possible mechanisms for conformational unmasking of critical neutralization epitopes may differ among them, in the present study we sought to test the hypothesis that these three proteins may induce synergistic or additive neutralization responses, such that combined immunization may result in more potent or cross-reactive neutralization.

## Methods

### Ethics Statement

The animal studies were carried out in strict accordance with the recommendations in the Guide for the Care and Use of Laboratory Animals of the National Institutes of Health. All efforts were made to minimize or avoid animal suffering. Rabbit bleeds were conducted with the use of anesthesia, and all procedures involving non-human primates were performed with the use of anesthesia. Research was conducted with approval of the Institutional Animal Care and Use Committees (Bioqual permit #10-3443-10 and Spring Valley Laboratories OLAW# is A3731-01).

The rhesus macaques (Macaca mulatta) used in this study were housed in accordance with the recommendations of the Association for Assessment and Accreditation of Laboratory Animal Care International Standards and with the recommendations in the Guide for the Care and Use of Laboratory Animals of the United States - National Institutes of Health. The Institutional Animal Use and Care Committee of BIOQUAL, approved these experiments. When immobilization was necessary, the animals were injected intramuscularly with 10 mg/kg of Ketamine HCl (Parke-Davis, Morris Plains N.J.). All efforts were made to minimize suffering. Details of animal welfare and steps taken to ameliorate suffering were in accordance with the recommendations of the Weatherall report, “The use of non-human primates in research”. Animals were housed in an air-conditioned facility with an ambient temperature of 21–25°C, a relative humidity of 40%–60% and a 12 h light/dark cycle. Animals were socially house when possible or individually housed if no compatible pairing could be found. The animals were housed in suspended stainless steel wire-bottomed cages and provided with a commercial primate diet and fresh fruit twice daily, with water freely available at all times. All animals survived the study in good health.

### HIV-1 *env* Genes

Most of the *env* clones used in production of pseudotyped viruses for neutralization testing and recombinant Env production have been described previously [48], [51]. *Env* used for pseudotyping viruses for neutralization assays were also obtained from the NIH AIDS Research and Reference Reagent Program Subtype B and C Neutralization Panels [51], [52]. Among those strains used from these panels, the subtype B strains 6535.3 and SS196 and the subtype C strain ZM1909F.PB4 are classified as Tier 1B based on neutralization sensitivity, the subtype B strain PVO.4 is classified as Tier 3, and the remaining strains are classified as Tier 2. The subtype A/E strains GX-E14, TH966, and E1035 have been previously described, and have not been classified with respect to neutralization sensitivity [48]. Deglycosylation mutants of the CM243 *env* were constructed for use in testing the hypothesis that gp41 glycosylation sites alter the ability of the Env to induce neutralizing antibodies. Mutations were introduced by site directed mutagenesis [37]. Asparagine-to-glutamine mutations were introduced at each of the four predicted glycosylation sites in gp41, singly or in selected combinations.

### Cells and Culture Conditions

Human HeLa (CCL-2), HeLaS3 (CCL-2.2), HuTk^-^ 143B (CRL 8303), HEK293T (CRL11554) cells, simian BSC-1 (CCL-26) and CV-1 (CCL-70) cells were obtained from the American Type Culture Collection, Manassas, VA. HuTk-, BSC-1 and CV-1 cells were maintained in Eagle’s Minimum Essential Medium supplemented with 10% bovine calf serum and 2 mM L-glutamine and antibiotics. HeLa cells were maintained in Dulbecco’s modified Eagle’s medium supplemented with 10% bovine calf serum (BCS) and 2 mM L-glutamine (DMEM-10) and antibiotics. HeLaS3 cells were maintained in vented spinner bottles at 37°C in Eagle’s minimum essential spinner medium, 5% horse serum, and 2 mM glutamine. HeLa cell monolayers were maintained in DMEM (Quality Biologicals, Gaithersburg, MD) supplemented with 10% BCS and 2 mM L-glutamine. Cell cultures were maintained at 37°C in a humidified 5% CO_2_ atmosphere. Roller bottle cultures of BSC-1 cells were maintained in EMEM-10 at 37° at 0.5 revolutions/minute.

### Venezuelan Equine Encephalitis Virus Replicon Particle Preparations

The pRepX VEE replicon vector was used for Env expression *in vivo*. The cloning of R2 strain gp160 and gp160ΔCT into this vector was described previously [44]. The *env*s encoding the gp160 sequences corresponding to strains 14/00/4 and CM243(N610Q) were cloned into pRepX following similar procedures [48]. VRP expressing each of these genes were prepared as described previously [44], [45]. Plasmids comprising the pRepX-Envs, pCV, and pGPm were used as templates for in vitro transcription of RNA, the RNAs were transfected into BHK cells by electroporation, VRP were harvested from media 2–3 days later, and infectivity determined by titration in BHK21 cells [44], [45]. The endpoint used for infectivity tritration was based on immunofluorescence testing for HIV-1 Env expression.

### Antibodies and Reagents

Polyclonal anti-gp140 rabbit antiserum (R2143) was prepared against purified and denatured IIIB gp140 (Dong et al, 2003). The monoclonal antibodies 17b, 48d, 23e and A32 were a gift from James E. Robinson, Tulane University Health Science Center, New Orleans, LA. The mAb CG10 was a gift from Jon Gershoni, Tel Aviv University. Four domain sCD4 was obtained from the AIDS Research and Reference Reagent Program, Division of AIDS, NIAID, NIH. M12, D12, D19, D25, D50, T50, T38, D61, D38, D54, T3,T4, T6, T9, T10, T18 and T30 were from a panel of 138 murine mAbs raised by immunization with soluble monomeric or oligomeric HIV-1, IIIB gp140 [53], [54].

### Recombinant gp140 Envs

The gp140 coding sequences of R2 (subtype B) and 14/00/4 (subtype F) were prepared by insertion of two translation termination codons following the lysine residue just prior to the predicted gp41 transmembrane domain, and arginine to serine substitutions at positions corresponding to residues 517 and 520 of the R2 Env to disrupt the protease cleavage site between the gp120 and gp41 subunits [2], [55]}. The gp140 *env* were subcloned into the vaccinia plasmid expression vector, pMCO2 linking them to a strong synthetic vaccinia virus early-late promoter [53]. Recombinant vaccinia viruses were prepared encoding the gp140 Envs R2 (vAC4) and 14/00/4 (vAC35) using standard methodology [53]. The gp140 Env construct for CM243 was prepared using the expression plasmid pCB53 which contains the full length *env* in pSC59 [56] by inserting two translation termination codons following the lysine residue just prior to the predicted gp41 transmembrane domain and used to generate recombinant vaccinia virus (vGK4). The predicted N-linked glycosylation sites at positions N610, N615, N624 and N636 in the CM243 coding sequence were removed in various combinations by substitution of a glutamine residue for the asparagine residues at those locations in both full-length Env encoding constructs and gp140 encoding Env encoding constructs. Combinations of N-linked glycosylation site mutations were prepared by mutating additional sites in an existing mutation containing construct as template. Recombinant vaccinia viruses designated vHC9, vHC10, vHC11, and vHC12 were prepared using the gp140 *env* CM243(N610/615/624/636/Q), CM243(N615/Q), CM243(N610/615/Q), and CM243(N610/Q), respectively.

Recombinant gp140 Envs were produced by infecting BS-C-1 cells with the various gp140 encoding recombinant vaccinia viruses and producing the proteins under serum-free conditions, and oligomeric fractions of the gp140 Envs were purified from culture supernatants using a combination of lentil lectin Sepharose 4B affinity and size exclusion chromatography as previously described [44], [45], [48].

### Cell-cell Fusion Assays

Differences in fusogenic activity between CM243 gp160 wild type and its N-linked glycosylation mutants were compared using a previously established β-galactosidase reporter gene cell-cell fusion assay [57]. Briefly, Hela cells (effectors) were transfected with 5 ug of CM243 wild type or mutant gp160 DNA using DOTAP (Boehringer Mannheim, Indianapolis, IN), incubated at 37°C for 4 hours and then infected with a recombinant vaccinia virus expressing T7 RNA polymerase (vTF7.3) overnight at 31°C. BSC-1 cells (targets) were infected with recombinant vaccinia virus expressing CD4 (vCB3), CCR5 (vHC1) and *LacZ* encoding reporter virus (vCB21R) overnight at 31°C. Effector cells and targets cells were then mixed at a ratio of 1∶1 (2×10^5^ total cells per well, 0.2-ml total volume) after overnight infection. After incubation with substrate, fusion was measured by calculating the rates of β-gal activity (change in optical density at 570 nm per minute×1,000).

### Metabolic Labeling and Immunoprecipitation

BS-C-1 cells (ATCC CCL-26) were infected with recombinant vaccinia viruses at an m.o.i. of 10 pfu/cell. At 6 hr post infection, the virus inoculum was replaced with methionine and cysteine-free MEM-2.5 (minimal essential medium and 2.5% dialyzed bovine serum) containing 200 µCi/ml of [^35^S] cysteine/methionine (Amersham) and incubated overnight. Immunoprecipitations were performed by incubating Env containing supernatants with 1–2 µg of mAb or 1 µl of polyclonal serum for 1 h at room temperature. 50 µl of 20% Protein G Sepharose bead suspension was then added and the samples were rotated for 1 h at 4°C. The beads were washed three times with 1 ml of Triton buffer (50 mM Tris-HCl, pH 8.0, 300 mM NaCl, 0.5% Triton X-100). Proteins were eluted in sample buffer at 100°C for 5 minutes and analyzed by 8% sodium dodecyl sulfate-polyacrylamide gel electrophoresis (SDS-PAGE), under reducing or non-reducing conditions and autoradiography.

### CD4 Induced Epitope Immunoprecipitation

Purified wild type and mutant gp140 glycoproteins (1 µg each) were incubated with or without excess of sCD4 (3 µg) for 1 hour at room temperature followed by 3 µg of mAb 17b, 48d, 23e or A32. Env complexes were immunoprecipitated with 50 µl of 20% Protein G Sepharose bead suspension and the samples were rotated for 1 h at 4°C. The beads were washed three times with 1 ml of Triton buffer (50 mM Tris-HCl, pH 8.0, 300 mM NaCl, 0.5% Triton X-100). Proteins were eluted in sample buffer at 100°C for 5 minutes and analyzed by 8% SDS-PAGE and Western blotting with an HIV-1 Env specific rabbit polyclonal antiserum R2143.

### Neutralization Assays

Neutralization assays were performed using Env-pseudotyped luciferase reporter viruses as previously described [36], [47], [48], [58]. Pseudotyped viruses were prepared by cotransfection of 293T cells with plasmids expressing respective *env* and pNL4–3.Luc.E^-^R^-^. Clarified, filtered supernatants were used in neutralization assays in HOS-CD4^+^CCR5^+^ cells, with luciferase activity as readout. Neutralization endpoints were the highest dilutions that resulted in ≥50% inhibition of luciferase activity compared to preimmune sera.

### Animals

The 36 adult Chinese rhesus macaques (*Macaca mulatta*) used in this study were captive-bred. Before their inclusion in the study, all animals were screened and confirmed to be free of antibodies to simian immunodeficiency virus, simian retrovirus, and simian T-cell leukemia virus type 1. The animals were housed at BIOQUAL, Inc., Rockville, MD, in accordance with American Association for Accreditation of Laboratory Animal Care (AAALAC) standards. Sera were collected from the femoral vein 10 days after each immunization. All procedures performed on macaques were performed under general anesthesia. Immunizations were administered subcutaneously in the upper hind leg. Doses of VRP-*env* administered were 10^6.5^ focus forming units (FFU) of the individual VRP preparations or a mixture of the three calculated to have a total of 10^6.5 ^FFU. Immunizations with gp140 were given in RiBi adjuvant or TiterMax (used for the last dose) per the manufacturer instructions. Doses administered contained 30 µg of monovalent gp140 or 10 µg each of the three proteins for the trivalent immunization in 0.5 ml total volume. Six monkeys were used per immunization group.

Adult New Zealand White rabbits were housed and cared for at Spring Valley Laboratories, Woodbine, MD, also in accord with AALAC standards. Sera were collected by bleed from the ear vein before the first vaccination and 10 days after each vaccination. Rabbits were treated with Ketamine and Xylene prior to bleeds. Terminal sera were collected by cardiac puncture under general anesthesia.

Immunizations were administered subcutaneously in the upper hind leg. Doses of VRP-*env* administered were 10^6.5^ focus forming units (FFU) of the individual VRP preparations or a mixture of the three calculated to have a total of 10^6.5 ^FFU. The rabbit gp140 immunizations were administered as 30 µg doses in 0.5 ml RiBi or TiterMax (used for the last dose) per the manufacturer instructions; RiBi formulated immunogens were administered at six 50 µl intradermal sites, 300 µl intramuscular into each hind leg, and 100 µl subcutaneous in the neck; TiterMax formulated immunogens were administered by intramuscular injection 0.5 ml into each hind leg. There were five groups of three animals each: the groups were wild type gp140 (CM243), N610/615/624/636Q, N615Q, N610/615Q and N610Q. A total of 4 immunizations were given at day 0, day 28, day 56 and day 198. A prebleed sample (10 ml) was collected on day 0, a test bleed (10 ml) was collected 7 days following the second injection (day 28), a crop bleed sample was collected 7 days following the third injection (day 56), and a crop bleed was collected 10 days following the forth injection (day 198). Control animals received 0.5 ml of adjuvant only at the same immunization schedule. The analysis of neutralizing activity of the various sera was carried out with sera collected following the final immunization.

### Serum Enzyme Immunoassay (EIA)

An antigen capture EIA was used to determine serum Ig responses. Immulon II plates (Dynex Technologies, Inc, VA) were coated with 100 µl/well of human HIV-1 immune globulin at 1∶1000 in PBS-N overnight at 4°C. After blocking with non-fat dry milk (Blotto) at 37°C for two hr, 100 ul of HIV-1 IIIB strain gp140, at a concentration of 50 ng/ml in Blotto with Tween were added to each well. The gp140 was purified from medium of cell cultures infected with recombinant vaccinia virus, as described [53]. The plates were incubated at 37°C for 1 hr. After washing, serially diluted monkey or rabbit sera were added to the wells, and the plates were incubated at 37°C for 1 hr. Reactions were further developed using biotinylated anti-rabbit or anti-human IgG. Positive and negative control sera were included in each assay. Sera were assigned titers equal to the highest dilutions that produced reactions twice the level of the negative control serum.

## Results

### CM243 Glycoprotein Expression

Previously, the immunogenicity of a soluble and secreted oligomeric Env, gp140, was investigated and the consequences of Env oligomerization on its antigenic structure was assessed through the generation and characterization of a large panel of Env-specific mAbs [53], [54]. A large fraction of anti-gp41 mAbs were noted to react against oligomer sensitive or specific epitopes. Some of these mAbs map to cluster I [59] and are contingent on Env’s quaternary structure. Some of these cluster I mAbs possess weak neutralization activity. In addition, these mAbs are also capable of reacting with native oligomeric Env on the surface of infected cells, and a recent study by another group has also indicated that cluster I epitopes in gp41 are well exposed in Env on virions and HIV-1 infected cells [60].

**Figure 1:**
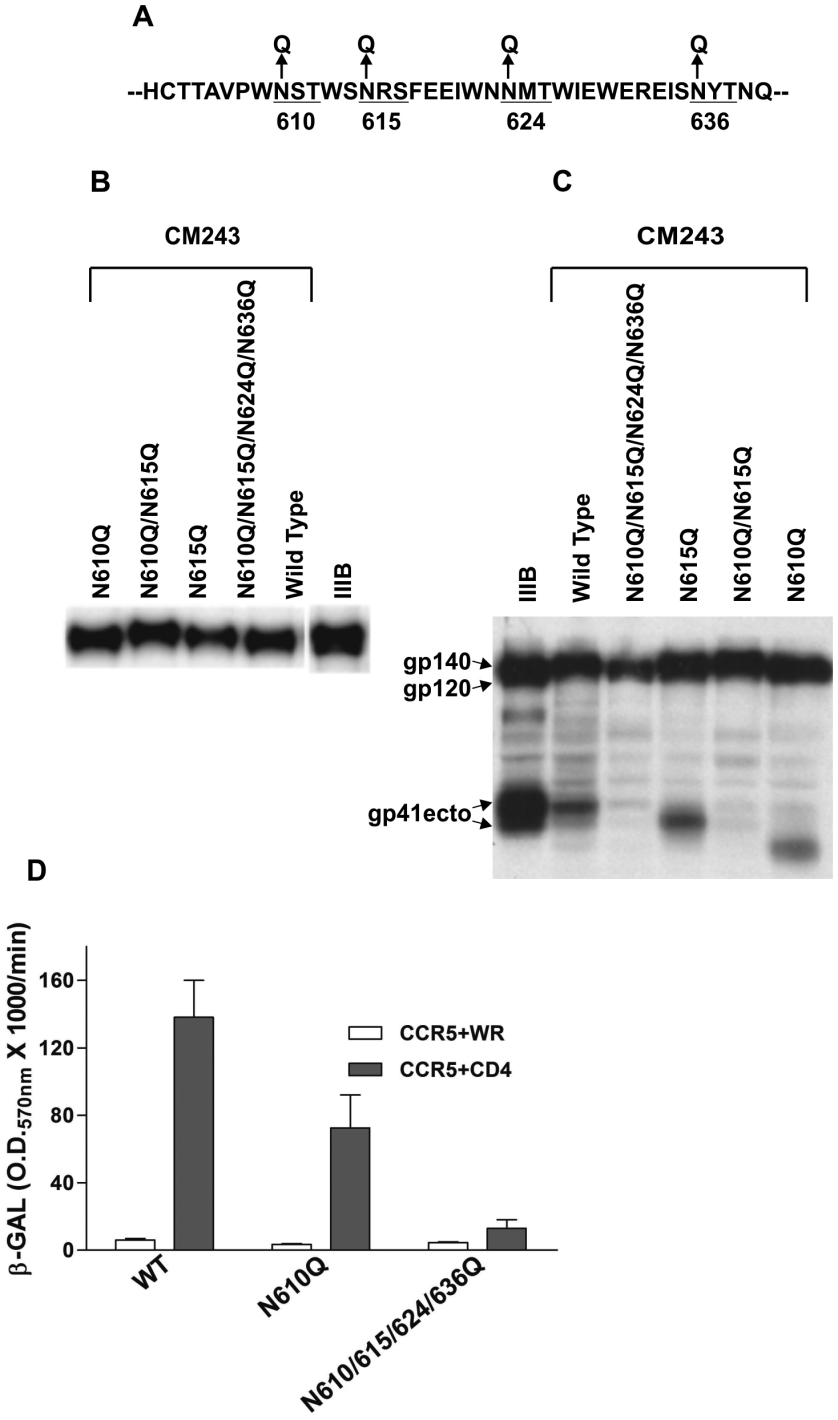
Deglycosylation mutants of HIV-1 strain CM243 gp140 Env. Panel A: Locations of point mutations in the gp41 ectodomain coding sequence that eliminate potential N-linked glycosylation sites. Panel B: Reduced SDS-PAGEanalysis of selected mutants of immunoprecipitated metabolically-labeled CM243 gp140 envelope glycoproteins using polyclonal rabbit antisera. Glycoproteins were transiently expressed in cells by plasmid transfection and infection with recombinant vaccinia viruses. Panel C: Analysis of precursor processing of metabolically-labeled CM243 gp140 wild type and glycosylation mutants by immunoprecipitation and reduced SDS-PAGEanalysis using a gp41 specific mAb (D61). Arrows point to gp41 ectodomains. Wild type CM243, single mutant N610Q and N615Q were processed to gp120 and gp41 ectodomain subunits. Where processing was evident the single N610Q resulted in the largest shift in apparent molecular weight of the gp41 ectodomain, an approximately 5–6 kDa loss. Panel D: Cell-cell fusion activity of selected CM243 mutants and wild type full length Envs using the β-gal reporter gene assay in transient transfected expressed constructs.

Here, we sought to extend these observations through the evaluation of a specific set of predicted N-linked glycosylation site deletion mutants in the context of an oligomeric HIV-1 gp140 Env. There are four highly conserved N-linked glycosylation sites in the gp41 ectodomain; one site is located in cluster I and the other three are located in cluster VI (Earl 1997). Our goal was to produce a set of modified oligomeric gp140 Envs and evaluate their immunogenic features with the hypothesis that removal of these conserved N-linked glycosylation sites in the gp41 ectodomain may enhance the elicitation of the oligomerization sensitive or specific, bcnAb reactivity previously observed. In preliminary experiments (not shown) CM243 *env* containing various predicted N-linked glycosylation site removal mutations were constructed in the context of both full-length and gp140 Envs. The sites in the gp41 coding sequence where the mutations were introduced are shown in **Figure 1A**. Expression was assessed by immunoprecipitation assay of culture supernatants from cells transiently transfected with expression plasmids and metabolic labeling using a rabbit polyclonal antibody R2143 and fusion function was determined using a reporter gene cell fusion assay. A subset of CM243 gp140 mutants which expressed protein at levels comparable to wild-type CM243 gp140 (N610Q, N615Q, N610/615Q and N610/615/624/636Q) (**Fig. 1B**) were chosen for further analysis and recombinant vaccinia viruses were generated for gp140 preparation. The expression and extent of Env processing of the CM243 gp140 glycoproteins produced by the recombinant vaccinia viruses was assessed by metabolic labeling and immunoprecipitation with the conformation-independent and cross-reactive gp41 specific mAb D61 (**Fig. 1C** **)**. The CM243 gp140 wild-type and mutants N615Q and N610Q were capable of some processing of the gp140 glycoprotein into the gp120 and gp41 ectodomain in comparison to HIV-1 IIIB gp140, and a shift in the mobility of the gp41 ectodomain in mutants N615Q and N610Q reveals their loss of N-linked glycosylation at those sites (**Fig. 1C**). Processing of mutants N610/615Q and N610/615/624/636Q was not evident. When these mutations were analyzed in the context of full length Env, all mutants were significantly impaired in their ability to mediate cell-cell fusion except CM243(N610Q) which exhibited fusion activity at a level approximately 50% of wild-type CM243 Env (**Fig. 1D**) which correlated with the processing data observed in the gp140 constructs.

### Immunoprecipitation of CM243 gp140 s by Murine mAbs

**Figure 2:**
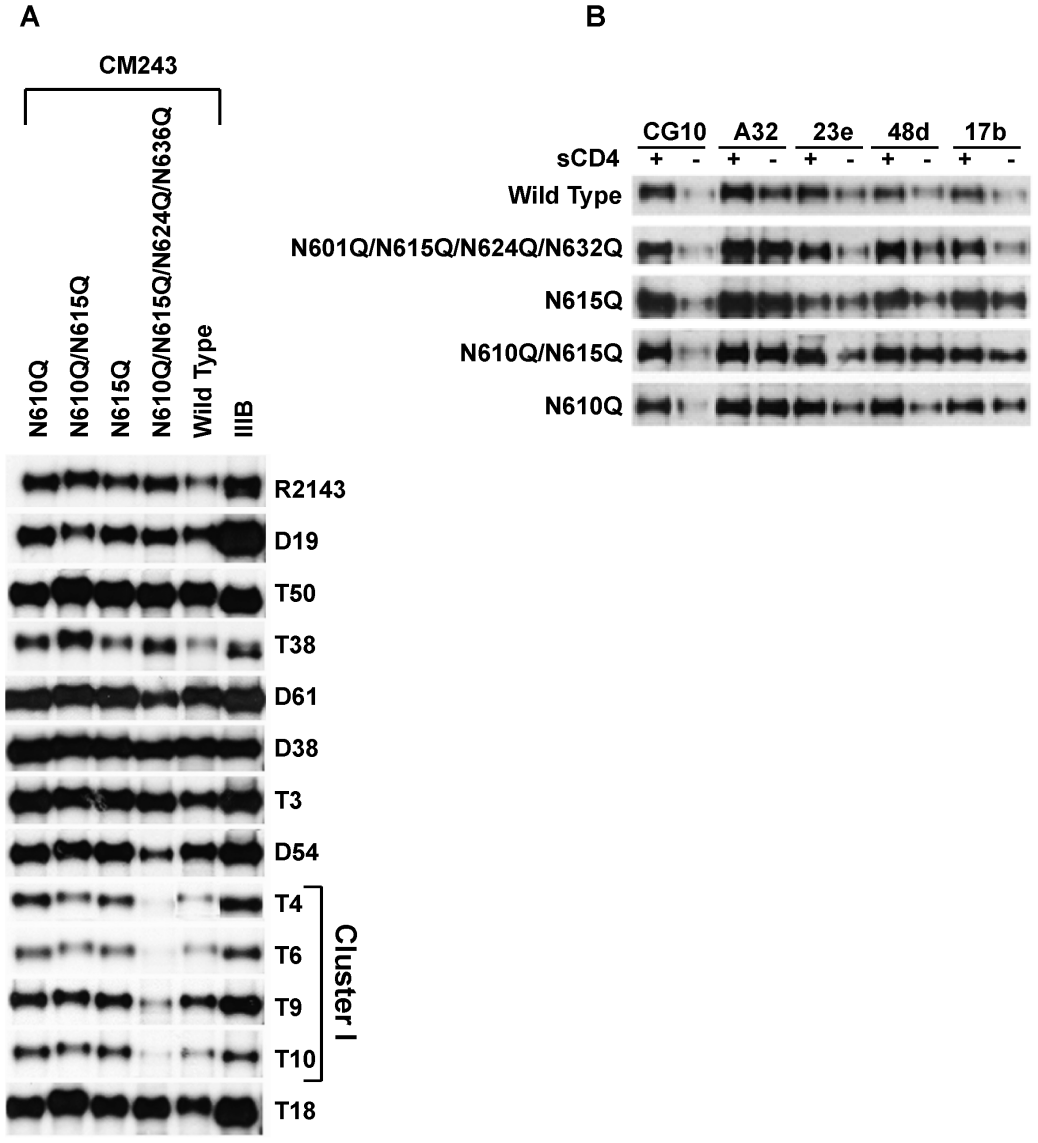
Monoclonal antibody and CD4 binding of the deglycosylation mutants of HIV-1 strain CM243 gp140 Envs. A. Reactivity of CM243 gp140 wild type and glycosylation mutants to a panel of conformation-dependent and -independent monoclonal antibodies by immunoprecipitation and reduced SDS-PAGE analysis of metabolically labeled gp140s and autoradiography. While equivalent reactivities with mAbs D19, T50, D38, and T3 were seen in both wild type and mutant gp140 s, differential reactivities were found with mAbs that map to the Cluster I region in gp41. These are conformation dependent, oligomeric specific anti-gp41 antibodies. B. Analysis of mAb binding to CD4i epitopes following sCD4 binding to purified gp140 proteins by immunoprecipitaion and SDS-PAGE and western blot detection using rabbit polyclonal antisera.

A panel of murine anti-gp41 mAbs raised against different forms of IIIB gp140 was used to characterize the deglycosylation mutants in immunoprecipitation assays (**Fig. 2A**) [56]. Excess amounts of gp140 supernatant were used in each of the immunoprecipitations. Among those mAbs that recognize conformation-independent epitopes (D19, T50, T36, D61, T3, D54, and T18), all bound approximately as well to each mutant gp140 in comparison to the wild-type. Among those mAbs that bind to oligomer-specific epitopes in Cluster I (mAbs T4, T6, T9, and T10), the binding of each to the quadruple mutant, gp140_CM243(N610/615/624/636Q)_, was reduced, while there was some indication that mAbs T4 and T10 reacted slightly better to CM243 mutants N601Q, N615Q and N610/615Q. These results suggested that the amino acid residue 624 and/or 636 mutations or the combination of three or four mutations resulted in conformational change that affected binding of mAbs to the Cluster I region, but that the mutations at residues 610 and 615 did not affect conformation in this manner, either alone or when combined. Effects on gp41 conformation outside Cluster I were not evaluated.

The inducibility of coreceptor binding region epitopes in response to CD4 binding in the CM243 gp140 glycoproteins was also assessed. Here, purified preparations of each CM243 gp140 were reacted with or without excess sCD4 followed by the addition of a CD4-inducible epitope (CD4i) mAb (3 µg). The immunoprecipitated gp140 s were then analyzed by SDS-PAGE and Western blotting with (**Fig. 2B**). There was similar effect of sCD4 on binding of CD4i mAbs to all of the mutants and wt Env, with the exception that the requirement for sCD4 may have been reduced in some cases for the Envs with mutations at residues 610 and 615.

### Comparative Immunogenicity of Wild-type and Deglycosylation Mutants of Strain CM243 gp140

**Figure 3:**
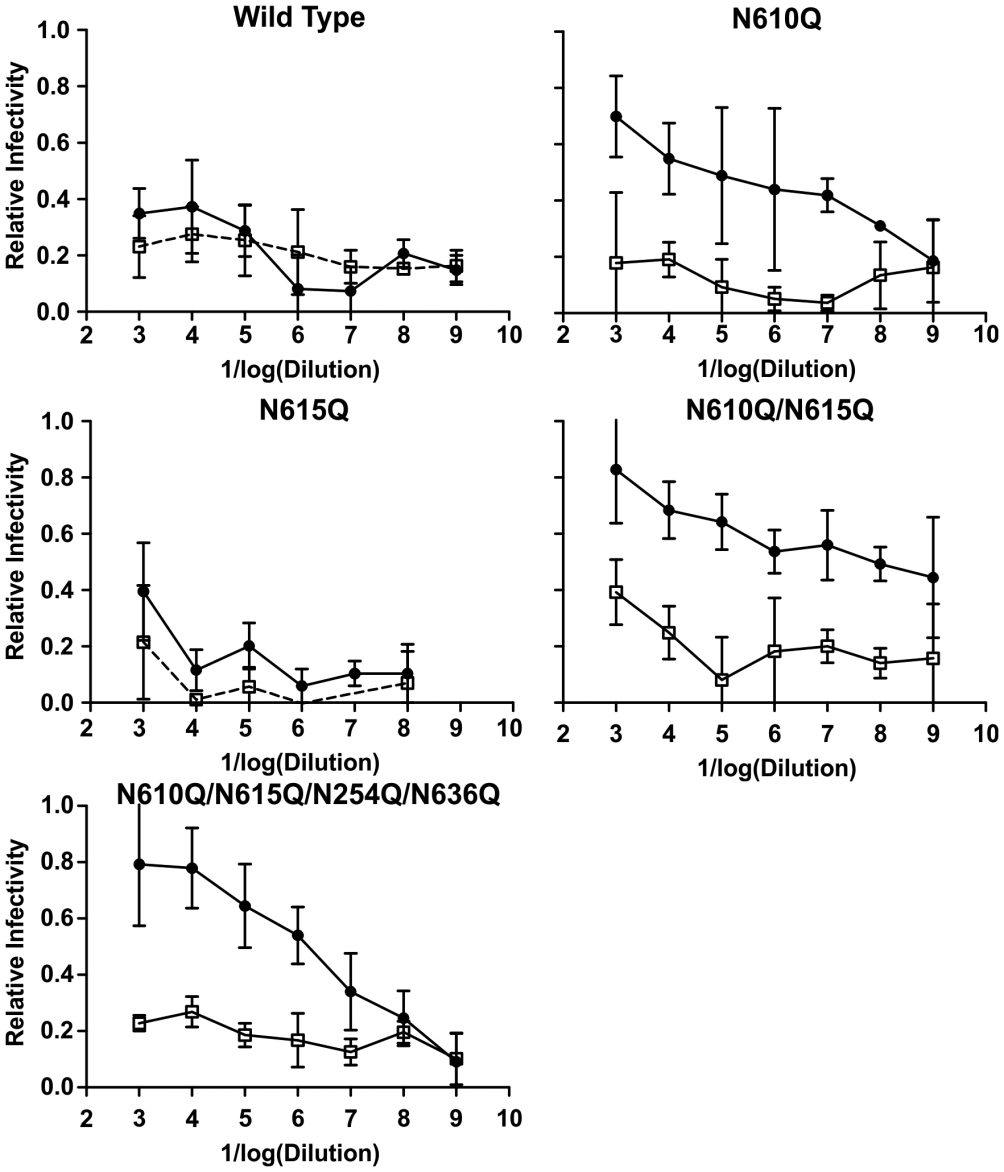
Induction of neutralizing antibody responses by mutant forms of CM243 Env containing deglycosylation mutations in NZW rabbits. There were five groups of three animals each: the groups were wild type gp140 (CM243), N610/615/624/636Q, N615Q, N610/615Q and N610Q. Rabbits were immunized four times with the indicated mutant gp140s. and sera were obtained for testing 2 weeks after the fourth doses. Sera were tested in serial two-fold dilutions for neutralization of virus pseudotyped with CM243 Env. Results obtained from the gp140 immunized rabbits are shown in black circles and for control (adjuvant only) rabbits in open circles. Results shown are means and standard deviations of three separate assays, each in triplicate, with sera collected following the final immunization.

Adult New Zealand White rabbits were inoculated with doses containing 30 µg of the respective gp140 in 0.5 ml RiBi adjuvant, or adjuvant alone, at 0, 3, 6, and 28 weeks. Sera were collected by bleed from the ear vein before the first vaccination and 10 days after each vaccination. The results of testing of sera from the post dose 4 bleed for neutralization of HIV-1 strain CM243 are shown in **Figure 3**. The inhibitory effect observed with the sera from the rabbits immunized with wild type CM243 strain gp140 was not different than control rabbit sera. The sera from the rabbits immunized with CM243(N610Q) gp140 also neutralized the HIV-1 strains R2, SF162, and 14/00/4 (all Tier 1 strains), while the control sera and sera from rabbits immunized with CM243 gp140 did not (not shown).

### Comparative Immunogenicity of Monovalent and Trivalent Immunization Regimens

**Figure 4:**
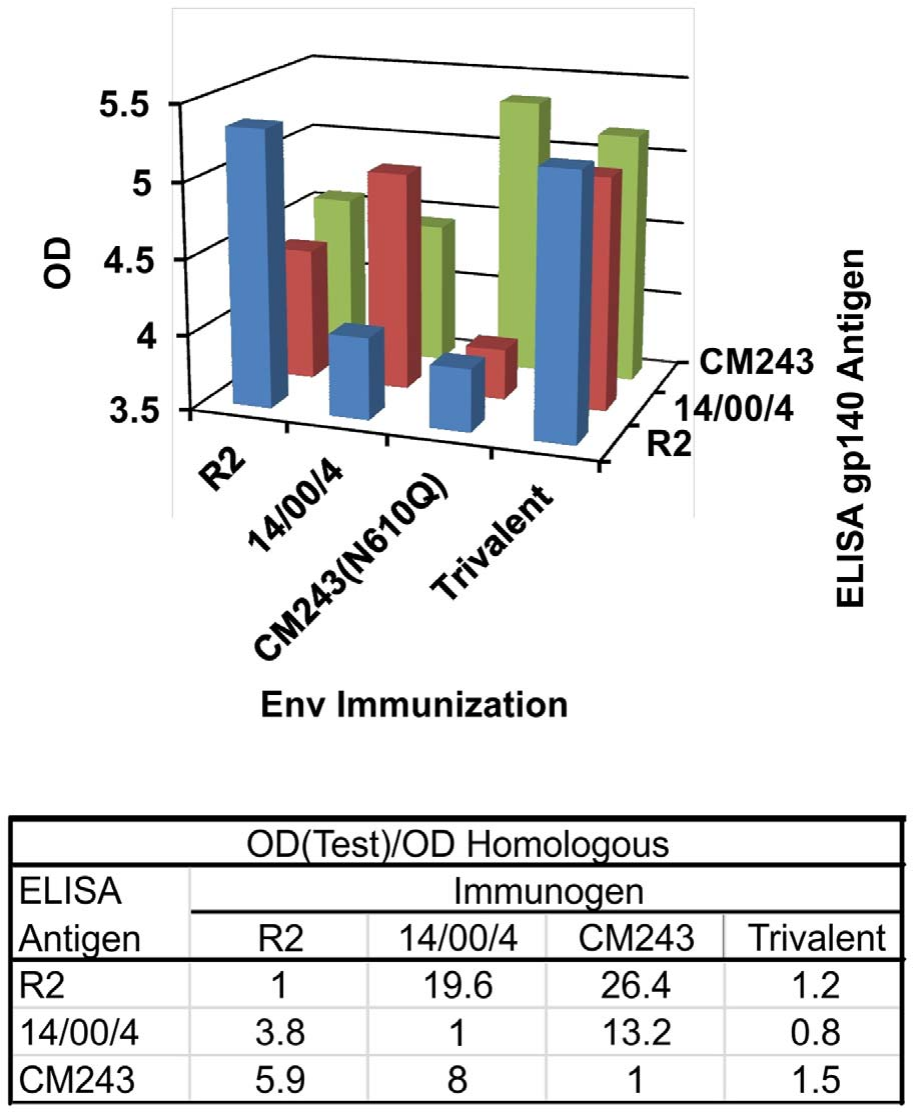
Immunoglobulin G responses of monkeys measured by enzyme linked immunosorbent assay using gp140 antigens of strains R2, 14/00/4, and CM243. A. Geometric mean titers are shown for the 6 monkeys in each of the immunization groups against each of the gp140 antigens. B. Ratios of GMT of sera of monkeys in group receiving the same antigen as the test/GMT of sera from monkeys receiving the heterologous antigens. Sera were substantially more reactive with homologous than heterologous antigens, and trivalent immunization resulted in responses against each antigen similar to homologous monovalent immunization.

We sought to compare the immunogenicity of different Envs with mutations potentially associated with enhanced immunogenicity and to determine whether simultaneous immunization with multiple such glycoproteins with distinctive properties would result in enhanced or synergistic neutralizing responses. Prior to initiation of this study we had obtained moderately cross-reactive neutralizing responses when the R2 Env was administered first in a series of doses of Venezuelan equine encephalitis virus (VEE) replicon particles (VRP) expressing the Env followed by a series of doses of soluble gp140 in RiBi adjuvant. The same immunization approach was used in the present study to compare the immunogenicity of the individual and combined glycoproteins. The study design is presented in **Table 1**. Doses of VRP expressing the respective gp160 s were given to monkeys at monthly intervals for three months, then at 4 monthly intervals for two more doses. Booster doses of respective gp140 s in RiBi adjuvant were given at three successive 3 month intervals. No further enhancement of response was observed after the last dose (data not shown), and no further immunizations were given. The results of antibody screening obtained by enzyme immunoassay (EIA) to Env antigens are shown in **Figure 4**. The sera were tested for binding to soluble gp140 proteins corresponding to the three strains used in the immunization protocol. For each antigen, the endpoint dilutions obtained were higher in sera from monkeys given homologous monovalent immunogens in comparison to those animals given the other monovalent immunogens. The differences between homologous and heterologous titers ranged from 3.3–32.4 fold. The antibodies detected in the sera from those monkeys given the trivalent immunogen preparation were similar to those measured in animals given homologous monovalent immunogen immunizations.

**Table 1:** Schedule for immunization of Rhesus Monkeys with VRP* Encoding gp160 Genes Followed by Booster Doses of gp140 in RiBi Adjuvant.

Study Group	Week			Month				
	0	4	8	6	10	13	20	24
I	VEE-R2	VEE- R2	VEE- R2	VEE- R2	VEE- R2	R2 gp14 + RiBi	R2 gp140 + RiBi	R2 gp140 + RiBi
II	VEE- 14/00/4	VEE- 14/00/4	VEE- 14/00/4	VEE- 14/00/4	VEE- 14/00/4	14/00/4 gp140 + RiBi	14/00/4 gp140 + RiBi	14/00/4 gp140 + RiBi
III	VEE- CM243 (N610Q)	VEE- CM243 (N610Q)	VEE- CM243 (N610Q)	VEE- CM243 (N610Q)	VEE- CM243 (N610Q)	CM243 (N610Q) gp140 + RiBi	CM243 N610Q) gp140 + RiBi	CM243 (N610Q) gp140 + RiBi
IV	VEE-R2+ 14/00/0+ CM243 (N610Q)	VEE-R2+ 14/00/4+ CM243 (N610Q)	VEE-R2+ 14/00/4+ CM243 (N610Q)	VEE-R2+ 14/00/4+ CM243 (N610Q)	VEE-R2+ 14/00/4+ CM243 (N610Q)	R2+ 14/00/4+ CM243 (N610Q) gp140s + RiBi	R2+ 14/00/4+ CM243 (N610Q) gp140s + RiBi	R2+ 14/00/4+ CM243 (N610Q) gp140s + RiBi
V	None	None	None	None	None	RiBi	RiBi	RiBi

* VEE-R2, VEE-14/00/4, and VEE-CM243(N610Q) indicate VEE replicon particle preparations (VRP) expressing the respective gp160 coding sequences. Monkeys received either the single VRP indicated, or all three (Group IV). Six monkeys per group were immunized at the indicated time points. Sera were collected approximately 14 days after each immunization for antibody testing.

**Figure 5:**
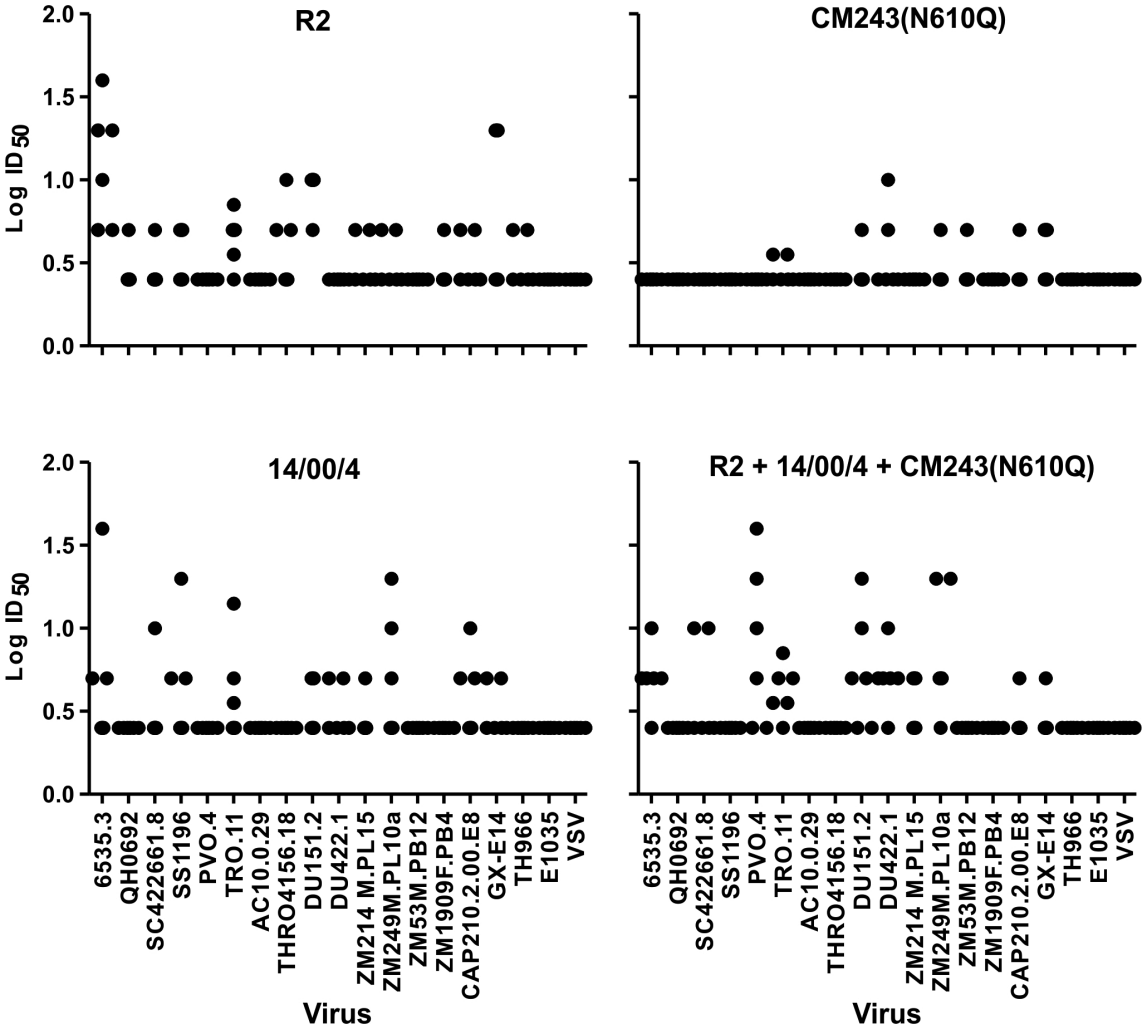
Neutralizing antibody responses of monkeys against viruses pseudotyped with subtype B, C, and A/E envelope glycoproteins. Sera were tested in serial dilutions starting at 1∶5. Sera with less than 50% neutralizing activity at 1∶5 were assigned inhibitory dilution 50% (ID_50_) of 2.5 (log dilution = 0.4). Dotted vertical lines indicate groupings of Clade B, C, and A/E pseudoviruses tested for neutralization.

The results of neutralizing antibody testing are shown in **Figure 5**. The data shown are means of two tests, each performed in triplicate. Each point represents the results obtained with serum from an individual monkey against a particular virus strain. Not unexpectedly, some weak inhibition was observed by sera from non-immunized monkeys in two cases, and in only one of two tests in each case, among 108 serum-virus test combinations (data not shown). Importantly, no neutralization was noted against the control vesicular stomatitis virus (VSV) G glycoprotein-pseudotyped lentivirus particles by sera from any monkeys. Neutralization ≥50% at a 1∶5 dilution was observed for at least one monkey serum in the R2 immunization group against 14 of the 18 virus strains tested, in the 14/00/4 group against 10/18 strains, in the CM243(N610Q) group against 7/18 strains, and in the trivalent group against 11/18 strains. The numbers of serum-virus combinations associated with neutralization ≥50% were 37, 24, 11, and 35 of 108 total per group for the R2, 14/00/4, CM243(N610Q), and trivalent immunogen groups, respectively. The number of serum-virus combinations associated with neutralization for all sera per group against all viruses differed significantly in comparisons of the R2 group to the 14/00/4 and CM243(N610Q) groups, the 14/00/4 group to the CM243(N610Q) group, and the CM243(N610Q) group to the Trivalent group. Thus, the data indicated that the R2 and trivalent immunogens induced similar responses, R2 was slightly more effective at inducing neutralizing antibodies than 14/00/4, and CM243(N610Q) was least immunogenic. We did not test the sera against other subtype F strains, so we do not know if the 14/00/4 regimen would have induced better subtype F neutralization than the other regimens.

The geometric mean neutralizing titers of the sera from the Group I monkeys, immunized with R2, against R2 and 14/00/4 strains were 1∶70.8 and 1∶28.2, indicating a 3.0-fold tendency for greater homologous neutralization that was not significantly different by paired T test (p = 0.10). The geometric mean neutralizing titers of the sera from the Group II monkeys, immunized with 14/00/4, against R2 and 14/00/4 strains were 1∶11.2 and 1∶89.1, indicating an 8.0-fold tendency for greater homologous neutralization that was significantly different by paired T test (p = 0.009). In comparisons between the groups, the R2 pseudotyped virus was neutralized by all sera from those monkeys immunized with the R2 Env, but by serum from only one monkey immunized with 14/00/4 at dilutions ≥1∶20, a difference that was significant by T test (p = 0.008). In contrast, the 14/00/4 Env pseudotyped virus was neutralized by 5/6 sera from monkeys immunized with R2 Env as well as all animals immunized with 14/00/4 Env at dilutions ≥1∶20, a difference that was not significant by T test (p = 0.11). Only two sera from the R2 group and one from the 14/00/4 group neutralized CM243(N610Q) virus at dilutions of 1∶20. Neutralization of R2 and 14/00/4 Env pseudtyped viruses by sera from Group IV monkeys (Trivalent immunization group) was comparable. The homologous neutralization results confirmed the somewhat greater breadth of response induced by R2 Env as compared to the 14/00/4 Env and the poorer responses induced by CM243(N610Q) Env; indeed, the CM243(N610Q) virus was generally resistant to neutralization by monkey sera.

## Discussion

The occurrence of bcnAb responses during natural infection does document that HIV-1 Envs can present epitopes to the immune system that induce antibodies that neutralize cross-reactively. The structural requirements for such Envs to induce those responses are of great interest and remain an important yet elusive goal of HIV-1 vaccine development.

In the present study, our goal was to determine whether simultaneous immunization with multiple HIV-1 Envs, selected individually on the basis of potential for induction of neutralizing antibodies, would result in induction of neutralizing antibody responses of enhanced potency and cross reactivity in comparison to immunization schemes with each Env alone. A secondary goal was to determine whether different specificities of neutralizing antibody responses would be obtained after immunization with the different Envs. We selected Envs of three different HIV-1 subtypes, each on the basis of differing evidence that they may be associated with a capacity to induce an effective antibody neutralization response. One particular Env, CM243(N610Q), was selected on the basis of results presented here which demonstrated that a mutation that eliminated an N-linked glycosylation site in gp41 proximal to the disulfide-bonded loop resulted in enhanced capacity to induce neutralizing antibodies in rabbits. The other two Envs were cloned from individuals possessing bcnAb responses to HIV-1 infection. The R2 Env has been reported to mediate CD4-independent infection and to induce a cross-reactive, albeit low potency, neutralizing antibody response [47], [48]. The Env 14/00/4, has an extremely rare mutation in the MPER of gp41, which conferred exceptional sensitivity to neutralization by mAbs directed against the region [61]. The immunogenicity of these Envs was studied both individually and in combination in rhesus macaques. As reported previously, the R2 Env induced neutralizing antibodies that were substantially cross-reactive among a group of neutralization resistant subtype B and C virus strains. The 14/00/4 Env induced neutralizing antibodies that were somewhat less cross-reactive than those induced by R2, and the CM243(N610Q) Env induced little neutralizing cross-reactivity among neutralization resistant strains. Immunization with the three Envs in concert did not induce more potent or cross-reactive neutralization than that induced by the R2 Env alone. These results did not support the hypothesis that immunization with multiple HIV-1 Envs would result in a more potent or cross-reactive neutralizing antibody response in comparison to immunization with a single Env subtype alone.

The potential for an enhanced capacity in the induction of neutralizing antibody responses as a result of deglycosylation mutations has been reported by some others, with respect to N-linked glycosylation sites in the gp120 subunit [62], [63]. However, this is the first study that examined effects of deglycosylation in gp41 in this regard. Ma et al studied effects of deglycosylation mutations in gp41 on antigenicity of gp140, and found that MPER epitopes were more exposed after deglycosylation based on binding to specific mAbs [64]. They also showed that deglycosylation of gp140 made it capable of inducing antibodies in monkeys that bind with greater affinity to MPER peptide, but did not assess neutralization. Preliminary studies of the immunogenicity of CM243 gp140 in RiBi adjuvant in rabbits did not demonstrate induction of any neutralizing antibody response, even against virus pseudotyped with homologous CM243 Env. Subsequently, the effects of various deglycosylation mutations on gp140 production in cell culture were examined. Substitutions predicted to eliminate N-linked glycosylation at the two sites closest to the disulfide-bonded loop in gp41 had no significant effect on protein secretion by cells or on reactivity in immunoprecipitation assays with various mAbs. Substitution at 3 or all 4 of the predicted glycosylation sites did result in reduced gp140 production. Since a murine mAb had been identified in a previous study that had weak neutralizing activity and bound very close to the gp41 disulfide-bonded loop, the effects of the two mutations closest to the loop, N610Q and N615Q, on induction of neutralizing antibody were studied. The potential for additive effects of multiple substitutions was studied by comparing the effects of each of those mutations separately and combined, as well as the quadruple mutant, N610Q/N615Q/N624Q/N636Q. We found that the N610Q mutant protein did induce neutralizing antibodies in rabbits, while the N615Q mutant did not, and the double and quadruple mutants were no more effective at inducing neutralizing antibodies than the single N610Q gp140 mutant. However, although unlikely, we cannot exclude the possibility that this effect could be due to the N to Q conversion itself and not an absence of N-linked glycosylation. Neutralizing activity was demonstrable against homologous virus and other sensitive laboratory HIV-1 Env strains. These results suggest that elimination of a potential N-linked glycosylation site in gp41 results in exposure of an immunogenic neutralization epitope in gp140. However, we have not been able to demonstrate increased sensitivity to neutralization of CM243(N610Q) by any cross neutralizing mAbs, and have no present hypothesis regarding the mechanism of this apparent increased immunogenicity.

The nonhuman primate immunization regimen used in the present study was similar to one we employed in prior studies, and when used for immunization with the R2 Env resulted in cross-reactive neutralizing responses and protection of monkeys against heterologous SHIV challenge [45]. Specifically, the immunization regimen consisted of administration of sequential doses of VRP expressing the Env gp160 sequences followed by sequential doses of purified soluble gp140 in RiBi adjuvant. We reasoned that this approach would result in the presentation to the immune system Envs with a conformation similar to the native conformation of functional Env spikes on virus particles. Env glycoprotein expressed in vivo on the surface of transduced cells should be similar to protein on the surface of virus particles and/or infected cells, and soluble gp140 with the gp120/gp41 cleavage site removed, as produced for this study includes both dimeric and trimeric oligomeric forms, as we have shown previously for the R2 gp140 Env protein [44], [45]. Further, the findings here indicate these mutant gp140 proteins do behave immunochemically much like intact or wild-type Env in their ability to bind a variety of mAbs, including CD4i reactive mAbs and CD4. Because of these characteristics of the immunization regimen, we anticipated that we may be able to induce antibody responses against conformational neutralization epitopes that were unmasked by deglycosylation, V3 or MPER mutations.

The gp140 binding antibody responses induced by each of the three monovalent immunization regimens were found similar. In each case the antibody binding activity against homologous gp140 Env glycoprotein was similar in magnitude, and significantly greater than binding against either of the other two gp140 Env proteins. Trivalent immunization resulted in antibody responses similar in binding potency for each of the three Env proteins to the homologous binding of the sera from those subjects that received the monovalent immunization scheme. Thus, it appeared that the three different monovalent immunization regimens were similar with respect to potency of IgG responses induced, and that the potency of the strain specific IgG responses induced by the trivalent regimen was similar to those induced by each of the monovalent regimens.

It is in some ways remarkable that there was no real evidence found of differential specificities of the neutralizing antibody responses induced by the different monovalent and trivalent immunization strategies. This interpretation is made in the sense that there was not a pattern in which one regimen induced neutralization that was relatively potent against one group of viruses tested, while other regimens induced responses that were active against other groups of viruses. While the primary viruses tested comprised only subtype B, C, and E Env strains, there did not appear to be different cross-reactive neutralization epitopes on these strains recognized differentially by responses induced by the different immunogens. Rather the magnitude of the responses differed with respect to neutralization potency and number of strains recognized. These data could also be consistent with the possibility that there is in fact a limited number of cross-reactive neutralization epitopes mediating cross-reactivity among these strains. Our data contrast somewhat with results reported by Wang et al in studies of gp120 immunization of rabbits [65]. They found more cross-reactive neutralization in rabbits that received multiclade mixtures of gp120 immunogens compared to monovalent immunogens. The difference between their results and ours could reflect exposure of different epitopes on gp120 and gp140/160, differences between strains of HIV-1, or other factors. Differences in potency of the responses induced by the different gp140 s could be related to differential capacity to engage germ line or minimally differentiated immunoglobulin receptors, as has been described for other gp140 s, but it is difficult to appreciate how such differences might be related to lack of differences in cross-reactivity of the neutralizing responses induced [66], [67].

Subsequent evaluation of the epitope specificity of the neutralizing responses induced in this study could lead to hypotheses regarding alternative approaches to multivalent immunization. For example if the R2 and 14/00/4 Envs induced antibodies that neutralized through recognition of the same epitope(s), alternative Envs could be selected that have been shown to induce antibodies that neutralize through recognition of different epitope(s). It is also possible that administration of different Envs sequentially instead of simultaneously would be more effective.

The goal of inducing highly potent and cross-reactive HIV-1 neutralizing antibodies remains elusive. The results presented here indicate that there was differential immunogenicity among HIV-1 Envs when administered in the regimen used. We did not find evidence that combining multiple Envs in an immunization regimen induced any greater responses than immunization with the one Env with the greatest immunogenicity. Alternative methods of inducing enhanced potency responses using HIV-1 Envs which induce antibody responses with adequate breadth of neutralization need to be explored.

### Conclusions

The study addressed three novel questions regarding requirements for induction of HIV-1 neutralizing antibodies. The importance of the four conserved glycosylation sites in gp41 were evaluated in studies of the subtype E Env, CM243, and demonstrated that protein secretion, processing into subunits, and antigenic reactivity could remain intact with mutation of the glycosylation site nearest to the disulfide-bonded loop at residue N610, but that other mutations or multiple mutations were not as well tolerated. The wild type CM243 Env did not induce homologous neutralizing antibodies in rabbits, when administered as soluble protein in adjuvant, but the N610Q mutant was immunogenic. Overall, the results indicate that the glycosylation sites in gp41 are important for structure and function, but that they may also be associated with conformational masking of neutralization epitopes.

There is little information published regarding comparative immunogenicity of different HIV-1 Envs, when administered in parallel. For this study we evaluated comparative immunogenicity of three very distinct Envs that were selected based on potential for immunogenicity. The R2 Env is subtype B, and is from a donor with bcnAb. It is unusual in that it has a V3 loop sequence that confers CD4-independence, and it has been demonstrated to induce cross-reactive neutralization in multiple small animal and non-human primate studies. The 14/00/4 Env is subtype F and is from a different donor with bcnAb. It is also unusual in that it has an exceedingly rare mutation in the MPER of gp41 that confers high sensitivity to neutralization by the mAb 2F5 and to CD4 binding site ligands. These two Envs were tested in parallel with the CM243(N610Q) Env in rhesus monkeys. The immunization regimen used was a prime-boost regimen that we have used previously with R2 Env, with induction of cross-reactive neutralization. The neutralizing response induced by R2 was the most cross-reactive of the three. The response induced by 14/00/4 was lesser, but similar, and may have appeared greater if subtype F strains were in our neutralization panel. Both of these bcn Envs were far superior to CM243(N610Q). The results clearly demonstrate that immunogenicity of Envs varies, and that certain Envs may be particularly immunogenic.

The third factor we evaluated was the potential for enhancement of the neutralizing response by coadministration of multiple Envs. The strategy involved use of Envs that were distinct with respect to subtype, as well as having differing, very distinct characteristics supporting hypothetical immunogenicity. The results did not indicate that trivalent immunization was superior in immunogenicity to the most immunogenic monovalent component of the regimen. While this result is disappointing in some respects, it also suggests that the number of cross-reactive neutralization epitopes may be sufficiently limited that multivalent immunogens may not be required for effective HIV-1 immunization.
